# The Relationship between Telomere Length and Mortality in Chronic Obstructive Pulmonary Disease (COPD)

**DOI:** 10.1371/journal.pone.0035567

**Published:** 2012-04-25

**Authors:** Jee Lee, Andrew J. Sandford, John E. Connett, Jin Yan, Tammy Mui, Yuexin Li, Denise Daley, Nicholas R. Anthonisen, Angela Brooks-Wilson, S. F. Paul Man, Don D. Sin

**Affiliations:** 1 The Providence Heart and Lung Institute at St. Paul's Hospital, The UBC James Hogg Research Centre & Department of Medicine, The University of British Columbia, Vancouver, British Columbia, Canada; 2 Division of Biostatistics, School of Public Health, University of Minnesota, Minneapolis, Minnesota, United States of America; 3 Faculty of Medicine, University of Manitoba, Winnipeg, Manitoba, Canada; 4 Canada's Michael Smith Genome Sciences Centre, British Columbia Cancer Agency, Vancouver, British Columbia, Canada; Leiden University Medical Center, The Netherlands

## Abstract

Some have suggested that chronic obstructive pulmonary disease (COPD) is a disease of accelerated aging. Aging is characterized by shortening of telomeres. The relationship of telomere length to important clinical outcomes such as mortality, disease progression and cancer in COPD is unknown. Using quantitative polymerase chain reaction (qPCR), we measured telomere length of peripheral leukocytes in 4,271 subjects with mild to moderate COPD who participated in the Lung Health Study (LHS). The subjects were followed for approximately 7.5 years during which time their vital status, FEV_1_ and smoking status were ascertained. Using multiple regression methods, we determined the relationship of telomere length to cancer and total mortality in these subjects. We also measured telomere length in healthy “mid-life” volunteers and patients with more severe COPD. The LHS subjects had significantly shorter telomeres than those of healthy “mid-life” volunteers (p<.001). Compared to individuals in the 4^th^ quartile of relative telomere length (i.e. longest telomere group), the remaining participants had significantly higher risk of cancer mortality (Hazard ratio, HR, 1.48; p = 0.0324) and total mortality (HR, 1.29; p = 0.0425). Smoking status did not make a significant difference in peripheral blood cells telomere length. In conclusion, COPD patients have short leukocyte telomeres, which are in turn associated increased risk of total and cancer mortality. Accelerated aging is of particular relevance to cancer mortality in COPD.

## Introduction

The pathogenesis of chronic obstructive pulmonary disease (COPD) is obscure. What is well known is that COPD is rare before 40 years of age even among heavy smokers and its incidence increases exponentially with aging. Furthermore, COPD frequently aggregates with other age-related co-morbidities such as osteoporosis, cardiovascular disease and dementia [1], [2]. Together, these data suggest that COPD is likely related to the aging process [3]. Telomeres are biomarkers of aging. Telomeres are DNA caps located at the end of chromosomes [4], protecting DNA against degradation and remodelling, and preventing gene-to-gene fusion or other gene rearrangements that may lead to cancerous changes [5], [6], [7]. Owing to the end-replication problem in mature somatic cells, telomere repeats are lost with each replicative cycle, until a critical length is reached at which point cells undergo apoptosis or other disruptive events [8]. This entire process is accelerated by the presence of reactive oxygen species (ROS) or inflammation [9], [10], [11], leading to short telomeres. Telomere length may thus be a robust indicator of human “biological age”. Previous studies in COPD suggest that telomeres of peripheral leukocytes are significantly shorter in COPD than in healthy control subjects [12], [13], [14]. However, the relationship of telomere length in peripheral leukocytes to important health outcomes such as mortality and cancer has not been explored in COPD. We hypothesized that COPD patients with longer telomeres would have a lower mortality rate than those with shorter telomeres and that telomere length serves as a predictor of mortality among COPD patients.

## Methods

### Subjects and Controls

**Lung Health Study (LHS).** The study used clinical data and biological materials obtained in the Lung Health Study (LHS), a clinical trial sponsored by the National Heart, Lung and Blood Institute. Following receipt of written informed consent, The LHS initially enrolled 5887 smokers, aged 35–60 years with mild to moderate airflow limitation (defined as a ratio of forced expiratory volume at one second (FEV_1_) to forced vital capacity (FVC)≤0.70 and 55<FEV_1_<90% predicted) across 10 centers in North America [15]. Individuals who had a history of cancer (except carcinoma in situ or basal cell carcinoma of the skin), myocardial infarction (in the past two years), angina, heart failure, stroke (in the past two years), renal failure, insulin-requiring diabetes mellitus, cirrhosis or other serious liver diseases, pulmonary embolism, disorders of the central nervous system, narrow-angle glaucoma, or any other major diseases which could have compromised follow-up were excluded [16].

During the first 5-years of follow up, the participants' lung function and smoking status were assessed yearly. Participants were categorized as sustained quitters if they were validated non-smokers at each annual visit. Participants who were smokers at each annual visit were continuing smokers. Those whose smoking behaviour varied were classified as intermittent quitters. At year 5, 4,803 participants provided blood samples (representing 89% of eligible participants). The blood samples were separated into their component parts and were stored in −70°C freezers until use. The study participants were then followed passively until the end of 2001 for a median follow-up of 7.5 years from the date of venipuncture to study closure. During follow-up, the participants' vital status and hospitalizations were recorded. A mortality and morbidity board reviewed all patient records including death certificates, eyewitness accounts, necropsy records, summaries of interviews with attending physicians and hospitalization records and assigned a cause to the mortality for all decedents. These data were supplemented by a National Death Index which provided the date and cause of death for all US study participants [16]. Mortality end points were the grouped into: coronary heart disease, cardiovascular disease (which also included coronary heart disease), lung cancer, all cancers (which included lung cancer), respiratory disease excluding lung cancer, others, and unknown.

**Healthy Aging Study Cohort.** To compare the telomere lengths of COPD subjects in LHS to a control group without COPD, we used telomere data from middle-aged subjects from the Healthy Aging Study, who were between 40 and 50 years of age and were randomly recruited without regard to health or disease status (i.e. “negative” controls) [17].

**Advair, Biomarkers in COPD (ABC) cohort.** As a second control group (i.e. “positive” controls), we measured telomere length of peripheral leukocytes obtained from patients with moderate to severe COPD, as defined spirometrically by FEV_1_/FVC ratio less than 70% and FEV_1_ less than 80% of predicted, who had at least 10 pack year smoking history and were at least 40 years of age [18]. The results of this study have been previously published [13].

### Leukocyte DNA Extraction

The concentration of DNA from peripheral blood collected at year 5 was determined using a NanoDrop 8000 spectrophotometer (Thermo Scientific, Wilmington, USA). The DNA samples were diluted to 1 ng/µL in 1× Tris-EDTA buffer and stored at −20°C for subsequent use in quantitative polymerase chain reaction (qPCR).

### Measurement of Telomere Length

Peripheral blood leukocyte telomeres were measured using a modified qPCR protocol described by Cawthon [19]. The primer sequences (written 5′→3′) were: tel 1, GGTTTTTGAG -GGTGAGGGTGAGGGTGAGGGTGAGGGT; tel 2, TCCCGACTATCCCTATCCCTATCCC -TATCCCTATCCCTA; 36B4u, CAGCAAGTGGGAAGGTGTAATCC; 36B4d, CCCATTCTA -TCATCAACGGGTACAA. The reference single copy gene used was 36B4 and the final concentrations of the primers (Sigma, The Woodlands, TX) were tel 1, 270 nM; tel 2, 900 nM, 36B4u, 300 nM; 36B4d, 500 nM.

Telomere length measurement was performed in triplicate for all the samples in a 384 well Clear Optical Reaction Plate (Applied Biosystems, Foster City, CA). Reference DNA obtained from the Coriell Institute (Camden, NJ) was assayed in triplicate on each PCR plate to account for interplate measurement variation. Each well contained 10 µL QuantiTect SYBR Green PCR Master Mix (QIAGEN, Mississauga, ON) and a final DNA concentration of 0.25 ng/µL. After loading, plates were sealed with MicroAmp Optical Adhesive Film (Applied Biosystems, Foster City, CA) and centrifuged briefly at 2,500 rpm. The reactions were performed in an ABI PRISM 7900HT Sequence Detection System (Applied Biosystems, Foster City CA). The thermal cycling profile for both telomere and single copy gene amplification began with 50°C for 2 min then 95°C for 2 min. For the telomere PCR, there followed 30 cycles of 95°C for 15 s and 54°C for 2 min. The 36B4 cycling profile was followed by 35 cycles of 95°C for 15 s and 58°C for 1 min. Telomere length was quantified as a relative T/S (T = telomere, S = single copy gene) ratio, calculated according to Cawthon's formula [19].

### Statistical Analysis

The telomere lengths of peripheral blood leukocytes were standardized to the reference single copy gene (T/S). For analytic purposes, the participants were divided into quartiles based on their T/S ratio. The clinical characteristics were then compared using a chi-square test for dichotomous variables (using appropriate degrees of freedom) and Cochran-Armitage test for trend for continuous variables. The primary endpoint for this study was all-cause mortality. We compared the risk of all-cause mortality across the quartiles over the follow-up period using a Kaplan-Meier (K-M) Method for the univariate analysis and a Cox proportional hazards model for the multivariate analysis. The K-M survival curves across the T/S quartiles were compared using a log-rank test. In the multivariate model, we included the following covariates: age, gender, body mass index (BMI), smoking status through the first 5 years of follow-up, pack-years of smoking, blood pressure at year 5, and FEV_1_ at year 5. FEV_1_, BMI, blood pressure measurements and pack-years of smoking did not significantly affect the results of the model. Thus, they were dropped in the final analysis. A similar approach was used for cause-specific mortality endpoints. T/S ratios across the smoking groups were compared using the Kruskal-Wallis test, as the data were non-normally distributed. Correlations between the T/S ratio and variables such as FEV_1_ at year 5 and age were tested using the Spearman test for non-parametric variables. P-values less than 0.05 (using a two-tailed test) were considered significant. All analyses were conducted using SAS (version 9.1, Carey, N.C.). The use of LHS and ABC study samples was approved by the Providence Health Care/UBC Research Ethics Board. The use of samples from the Healthy Aging Study was approved by the joint Clinical Research Ethics Board of UBC and the British Columbia Cancer Agency.

## Results

### Clinical Characteristics of the Participants

**Figure 1:**
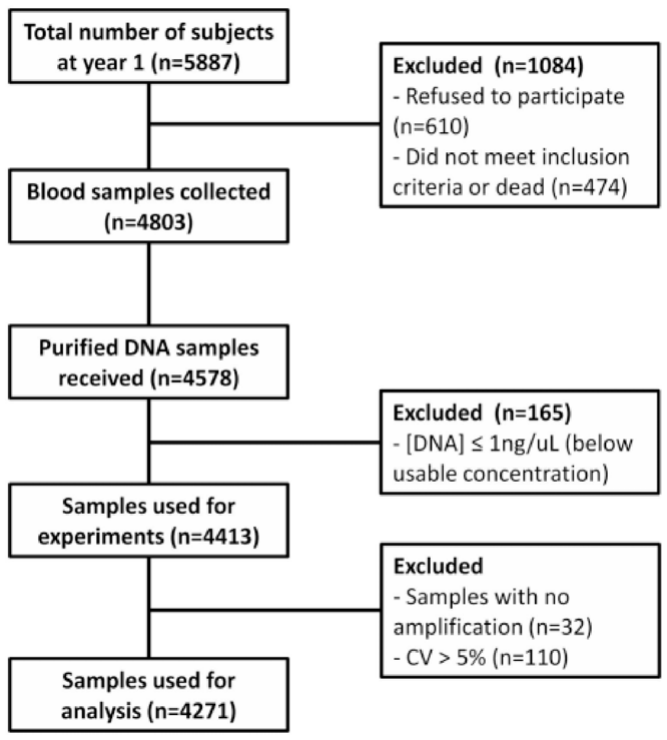
The number of participants excluded from analyses and reasons for exclusion. At year five, 5413 participants were alive and were eligible for venipuncture. Of those, 4803 provided blood samples (89% of eligible participants). DNA was extracted and purified from the buffy coat samples from a total of 4578 participants. Samples with concentration below 1 ng/uL were excluded and 4413 samples were used for telomere length measurement. Samples with greater than 5% coefficient of variation (CV) and with no amplification were excluded. Data analyses were carried out using telomere length measurements of 4271 subjects.

We successfully purified 4,578 genomic samples from the LHS. Samples (n = 165) with concentrations lower than 1 ng/µL were excluded from the analysis. Samples with a high coefficient of variation (>5%) in relative T/S ratio and those that did not amplify were also excluded, leaving 4,271 samples (93.3%) for the final analysis (**Figure 1**). Table 1 shows the main clinical characteristics of the LHS participants according to the relative length of peripheral blood leukocyte telomeres.

**Table 1:** Clinical and demographic features of the LHS participants, by quartiles of leukocyte telomere length, in the LHS (N = 4271).

Characteristic	Quartile				P value for trend
	1	2	3	4	
**Number of subjects**	1068	1068	1068	1067	
**Age (years)**	53.9±6.7	53.4±6.7	53.3±6.9	53.4±6.7	0.145
**Gender (% male)**	62.3	64	62.6	62.6	0.965
**BMI**	25.5±4.0	25.5±3.8	25.6±3.8	25.6±4.0	0.639
**Race (% Caucasian)**	96.7	96.9	95.5	96.0	0.157
**Continuous smokers (%)**	51.2	55.8	56.9	53.6	0.850
**Intermittent smokers (%)**	30.6	28.1	26.6	26.4	
**Sustained quitters (%)**	18.2	16.1	16.5	20.0	
**Pack years**	40.2±18.0	40.2±18.0	39.6±18.4	39.7±19.0	0.194
**FEV_1_(% predicted) at baseline**	78.4±8.8	78.7±8.7	78.3±9.9	78.4±10.6	0.415
**FEV_1_ (% predicted) after 5 years**	75.2±11.6	75.4±11.9	74.4±12.7	75.6±12.6	0.486

Abbreviations: FEV_1_, forced expiratory volume in 1 second; BMI, body mass index. Study participants were divided into 4 identical groups based on the telomere length, 1^st^ quartile being the shortest and 4^th^ quartile being the longest. Data are presented as mean ± SD and tested with Cochran-Armitage test for trend.

### Telomere Length Association with Baseline Characteristics

There were no significant differences in age, sex, BMI, race or cumulative smoking exposure (pack-years) across the quartiles of T/S ratio (**Table 1**). The median (relative) telomere length (T/S ratio) was 0.3256 (interquartile range: 0.2124 to 0.5000). Telomere length was non-normally distributed with no significant differences between males (n = 2685; median = 0.3246; interquartile range, 0.2133–0.4987) and females (n = 1586; median = 0.3268; interquartile range, 0.2102–0.5016). There was no significant relationship between the T/S ratio and age (r = −0.020; p = 0.195), FEV_1_% predicted (r = 0.001; p = 0.946) or BMI (r = 0.011; p = 0.474).

### Mortality Risk Prediction by Telomere Length

**Figure 2:**
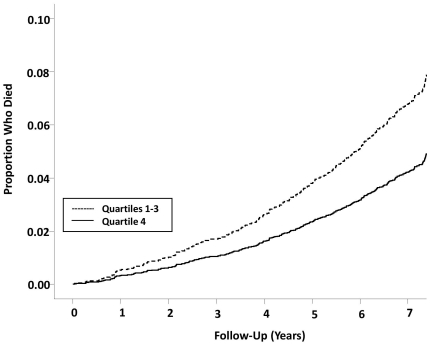
Kaplan-Meier survival curves for all-cause mortality by leukocyte telomere length of 4271 subjects in the LHS. Subjects are divided into quartiles 1, 2, and 3 (shorter telomeres) versus quartile 4 (longest telomeres). P value was derived from multivariable Cox models. Hazard ratio between Q1–3 and Q4 is 1.48 (95% CI, 1.02–1.66).

During a median follow-up of 7.51 years (interquartile range, 7.41 to 7.58 years) from blood collection to 2001, 399 of 4271 participants (9.34%) died; 202 from cancer (4.73%), 127 from lung cancer (2.87%), 52 from cardiovascular disease (1.22%) and 27 from non-malignant respiratory causes (0.867%). The T/S ratio was significantly related to all-cause mortality but the risk was non-linear (Table 2). The risk of all-cause mortality was similar across the first 3 quartiles (p>0.05) of T/S ratio but dropped significantly in the 4^th^ quartile (hazard ratio, HR, 1.30; p = 0.0331 comparing 1^st^ to 3^rd^ quartiles to 4^th^ quartile). Adjustment for covariates made no significant differences to the overall results (adjusted HR, 1.29; p = 0.0425). These data were largely driven by cancer mortality (**Table 2**). Compared to individuals in the 4^th^ quartile of T/S ratio, the remaining participants had significantly higher risk of cancer mortality (adjusted HR, 1.48 p = 0.0324). The hazard ratio of lung cancer mortality failed to reach significance and this may be due to the relatively small number of lung cancer mortality cases during follow-up (adjusted HR, 1.48; p = 0.0886). The K-M survival analyses of all-cause mortality and cancer mortality are shown in **figures 2** **and** **3**, respectively.

**Figure 3:**
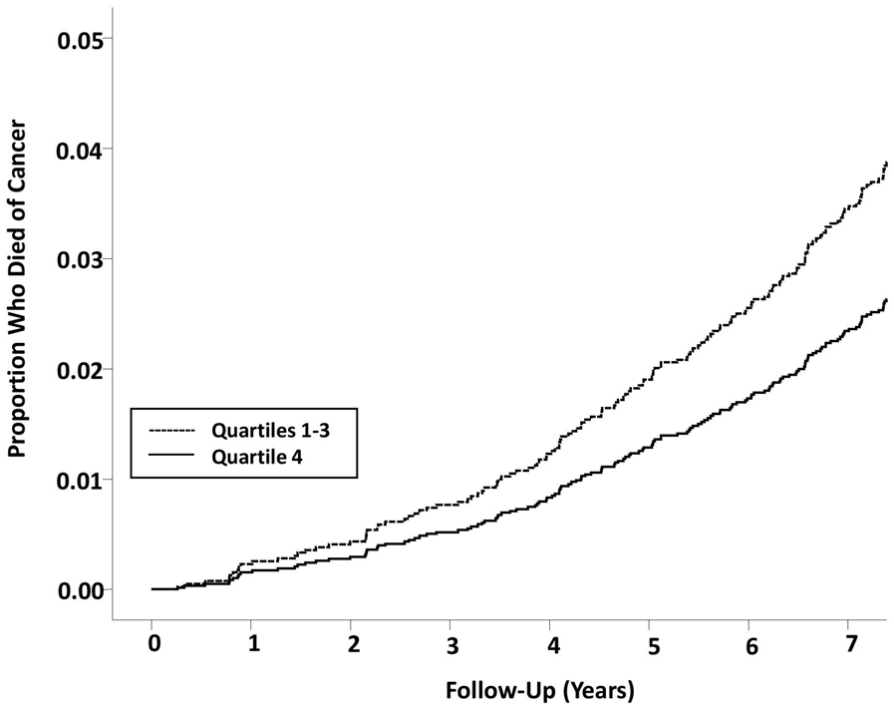
Kaplan-Meier survival curves for cancer mortality by leukocyte telomere length of 4271 subjects in the LHS. Subjects are divided into quartiles 1, 2, and 3 (shorter telomeres) versus quartile 4 (longest telomeres). P value was derived from multivariable Cox models. HR between Q1–3 and Q4 is 1.48 (95% CI, 1.04–2.11).

**Table 2:** Hazard ratio of total and disease specific causes of mortality according to telomere length.

End Point	1^st^ vs 4^th^ quartile	2^nd^ vs 4^th^ quartile	3^rd^ vs 4^th^ quartile	1–3 versus 4^th^ quartile
**Total Mortality**	1.20 (0.90, 1.61)	1.18 (0.88, 1.59)	1.48 (1.12, 1.96)	1.29 (1.01, 1.64)
**Cancer Mortality**	1.43 (0.94, 2.16)	1.35 (0.88, 2.07)	1.65 (1.10, 2.49)	1.48 (1.04, 2.11)
**Lung Cancer Mortality**	1.40 (0.83, 2.38)	1.53 (0.91, 2.60)	1.51 (0.89, 2.56)	1.48 (0.94, 2.33)
**CVD Mortality**	2.09 (0.85, 5.12)	1.95 (0.79, 4.84)	2.19 (0.90, 5.33)	2.08 (0.94, 4.61)
**Respiratory Mortality**	1.69 (0.57, 5.06)	1.59 (0.52, 4.86)	3.02 (1.10, 8.32)	2.09 (0.81, 5.36)

Adjustments were made for age, sex, smoking status (i.e. sustained smokers, intermittent quitters, quitters). Inclusion of other variables such as FEV_1_% predicted, body mass index and pack years of smoking made no significant differences to the results.

### Telomere Length and Smoking Status

Because cigarette smoke is suggested to accelerate telomere attrition, we evaluated the effect of smoking status on leukocyte telomere length. There was no significant difference in telomere length between the quitter group and the smoker groups (sustained quitters versus intermittent quitters: p = 0.074; sustained quitters versus continuous smokers: p = 0.605).

**Figure 4:**
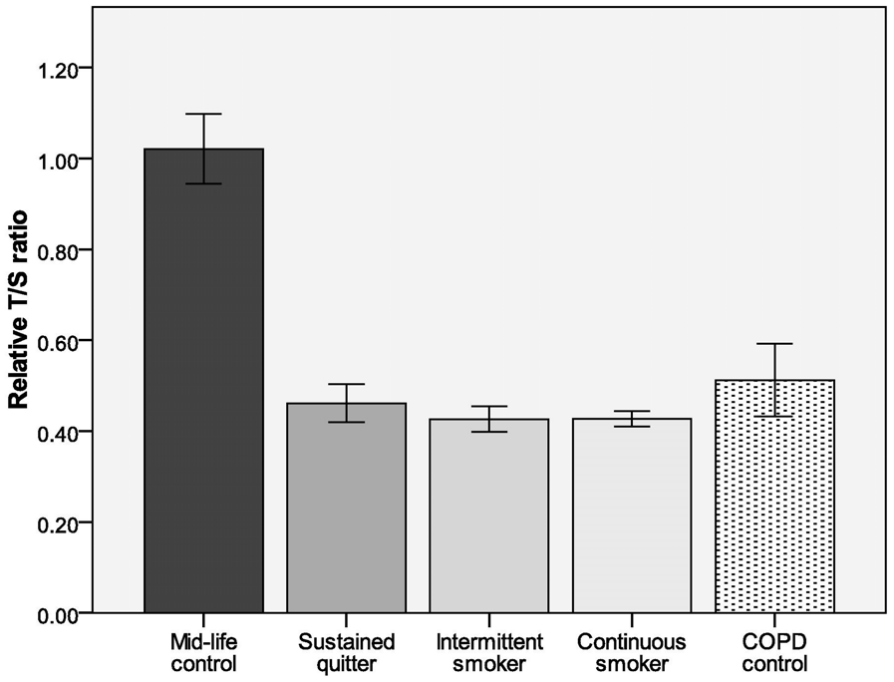
Telomere length in smoker groups in LHS and mid-life controls. Mid-life controls (n = 53; mean age = 45.34), sustained quitters (n = 753; mean age = 54.07), intermittent quitters (n = 1,192; mean age = 53.26), continuous smokers (n = 2,319), COPD (ABC) controls (n = 20; mean age = 64.80). Results are expressed as mean ± standard error. *p<0.001 vs. mid-life control group.

We also compared telomere length of various smoker groups in the LHS to the mid-life comparison group (mean age±SE: 45.34±0.51). The telomere length of the mid-life controls was significantly longer than all three smoker groups in the LHS (sustained quitter: p<0.001; intermittent quitter: p<0.001; continuous smoker: p<0.001) (**Figure 4**). The significant difference persisted when controlled for age.

Because the mid-life control participants' samples were collected more recently compared to the LHS genomic samples, ABC study samples were also compared to rule out telomere perturbation related to long-term storage of blood samples. There was no significant difference in leukocyte telomere length between LHS and ABC subjects (p = 0.4820), suggesting that storage condition is not the main driver of the difference between mid life controls and LHS samples shown in **Figure 4**.

## Discussion

The most important and novel finding from our study was that the telomere length of peripheral blood leukocytes of patients with COPD was significantly related to the risk of all-cause and cancer mortality over a median follow up of 7.5 years, independent of possible confounders such as chronological age, smoking status and lung function. Interestingly, the relationship was non-linear with the lowest risk observed in the group with the longest T/S ratio, suggesting a threshold effect. We also found that although smoking status was not associated with differential telomere lengths, the telomere length of peripheral leukocytes from LHS participants, who all had significant smoking history (on average more than 40 pack-years) at the time of blood collection, was significantly shorter (by more than 50%) than that of healthy subjects of similar age. Together, these data suggest that prior cigarette exposure (or COPD) accelerates telomere attrition, leading to short telomeres and, in its extreme, short telomeres are associated with increased risk of cancer and total mortality in COPD patients.

The mechanism that underlies the association between short telomeres in leukocytes and mortality in COPD patients is unknown. The first possibility is that oxidative stress from environmental oxidants (cigarette smoke) and chronic inflammation leads to accelerated shortening of telomeres, and predisposes COPD patients to adverse outcomes including death from cancer. However it is also possible that COPD patients who had an adverse health outcome were genetically predisposed to accelerated telomere shortening. The above explanations are not mutually exclusive, as shorter telomeres in leukocytes of COPD patients that develop cancer might reflect both constitutionally short telomeres and further shortening by oxidative damage.

One of the strengths of this study was its longitudinal design. The cohort was made up of mild to moderate COPD patients, who were passively followed for a significant amount of time, providing a unique opportunity to examine the relationship of telomere lengths to clinical outcomes in COPD patients. Another strength of this study was the large sample size. Previous clinical studies examining the relationship between telomere length and incidence of COPD have demonstrated mixed results owing largely to small sample sizes [14], [20].

There were also some limitations to the present study. First, although the qPCR method of quantifying telomere length is well accepted and widely used (for high throughput analysis), its signal to noise ratio is still relatively low [21], which may have led to an underestimation of the relationship between T/S ratio and adverse outcomes in our study. Nevertheless, it was reassuring that the qPCR methods produced similar results to those produced by flow FISH (data not shown; available from authors upon request). Second, most of the patients in this study had mild to moderate disease; thus, the relationship of T/S ratio to health outcomes across the full range of COPD severity is unknown. This may also explain the lack of significant association between T/S ratio and lung function in our study. Furthermore, all of the LHS participants had heavy smoking exposure history before blood samples were collected (on average more than 40 pack-years of smoking), which may have obfuscated the relationship between smoking cessation (status) and T/S ratio in our study. Third, the LHS did not have biological materials from lungs. Thus, the relationship of T/S ratio in peripheral blood leukocytes to that in lung tissues is uncertain. Lastly, telomere length was measured using genomic samples taken from buffy coat which also contains terminally differentiated cells and we did not have serial measurements from the same subjects before and after smoking cessation. Thus, it is uncertain whether telomeres in peripheral blood cells can “recover” following smoking cessation or whether it is an irreversible, progressive process.

Interestingly, we did not find a significant relationship between chronological age and T/S ratio. While the exact reason for this unclear, this finding is consistent with previous studies, which have shown that the relationship between chronological age and T/S ratio is significantly mitigated by cigarette smoking and by the presence of COPD. In Savale et al. [14], for instance, in the control population, there was a strong relationship between chronological age and T/S ratio (p = .0003). However, in patients with COPD, this relationship was significantly weakened (p = 0.42). Smoking also reduces T/S ratio [22], [23] and attenuates the relationship between chronological age and T/S ratio [22], [23]. In the Alpha-Tocopherol, Beta-Carotene Cancer Prevention (ATBC) Study, which similar to our study in that it evaluated only smokers, there was no significant relationship between chronological age and T/S ratio [22].

In summary, we have shown that COPD patients who have reduced telomere lengths in their peripheral blood leukocytes are at higher risk of total and cancer-related mortality. Our results are consistent with previous studies demonstrating a link between reduced telomeres and increased risk of cancer mortality in non-COPD elderly populations [24]. COPD is a worldwide epidemic and is projected to increase as smoking frequencies rise and the population ages [25]. Our results indicate that some COPD patients have short telomeres. This may be a distinct phenotype that is associated with increased risk of cancer and total mortality. Since telomeres can be readily measured in the peripheral circulation, telomeres are a promising biomarker in identifying this phenotype. However, given the variation in the T/S data, additional studies will be needed to address the impact of this measurement on clinical outcomes of COPD.

## References

[1] Mapel DW, Hurley JS, Frost FJ, Petersen HV, Picchi MA (2000). Health care utilization in chronic obstructive pulmonary disease. A case-control study in a health maintenance organization.. Arch Intern Med.

[2] Soriano JB, Visick GT, Muellerova H, Payvandi N, Hansell AL (2005). Patterns of comorbidities in newly diagnosed COPD and asthma in primary care.. Chest.

[3] Lee J, Sandford A, Man P, Sin DD (2011). Is the aging process accelerated in chronic obstructive pulmonary disease?. Curr Opin Pulm Med.

[4] Moyzis RK, Buckingham JM, Cram LS, Dani M, Deaven LL (1988). A highly conserved repetitive DNA sequence, (TTAGGG)n, present at the telomeres of human chromosomes.. Proc Natl Acad Sci U S A.

[5] Harley CB (1991). Telomere loss: mitotic clock or genetic time bomb?. Mutat Res.

[6] Levy MZ, Allsopp RC, Futcher AB, Greider CW, Harley CB (1992). Telomere end-replication problem and cell aging.. J Mol Biol.

[7] Munoz P, Blanco R, Blasco MA (2006). Role of the TRF2 telomeric protein in cancer and ageing.. Cell Cycle.

[8] Capezzone M, Cantara S, Marchisotta S, Filetti S, De Santi MM (2008). Short telomeres, telomerase reverse transcriptase gene amplification, and increased telomerase activity in the blood of familial papillary thyroid cancer patients.. J Clin Endocrinol Metab.

[9] Hayflick L, Moorhead PS (1961). The serial cultivation of human diploid cell strains.. Exp Cell Res.

[10] von Zglinicki T (2002). Oxidative stress shortens telomeres.. Trends Biochem Sci.

[11] von Zglinicki T, Saretzki G, Docke W, Lotze C (1995). Mild hyperoxia shortens telomeres and inhibits proliferation of fibroblasts: a model for senescence?. Exp Cell Res.

[12] Houben JM, Mercken EM, Ketelslegers HB, Bast A, Wouters EF (2009). Telomere shortening in chronic obstructive pulmonary disease.. Respir Med.

[13] Mui TS, Man JM, McElhaney JE, Sandford AJ, Coxson HO (2009). Telomere length and chronic obstructive pulmonary disease: evidence of accelerated aging.. J Am Geriatr Soc.

[14] Savale L, Chaouat A, Bastuji-Garin S, Marcos E, Boyer L (2009). Shortened telomeres in circulating leukocytes of patients with chronic obstructive pulmonary disease.. Am J Respir Crit Care Med.

[15] Anthonisen NR, Connett JE, Kiley JP, Altose MD, Bailey WC (1994). Effects of smoking intervention and the use of an inhaled anticholinergic bronchodilator on the rate of decline of FEV1. The Lung Health Study.. JAMA.

[16] Anthonisen NR, Skeans MA, Wise RA, Manfreda J, Kanner RE (2005). The effects of a smoking cessation intervention on 14.5-year mortality: a randomized clinical trial.. Ann Intern Med.

[17] Halaschek-Wiener J, Vulto I, Fornika D, Collins J, Connors JM (2008). Reduced telomere length variation in healthy oldest old.. Mech Ageing Dev.

[18] Sin DD, Man SF, Marciniuk DD, Ford G, FitzGerald M (2006). Can inhaled fluticasone alone or in combination with salmeterol reduce systemic inflammation in chronic obstructive pulmonary disease? Study protocol for a randomized controlled trial [NCT00120978].. BMC Pulm Med.

[19] Cawthon RM (2002). Telomere measurement by quantitative PCR.. Nucleic Acids Res.

[20] Morla M, Busquets X, Pons J, Sauleda J, MacNee W (2006). Telomere shortening in smokers with and without COPD.. Eur Respir J.

[21] Horn T, Robertson BC, Gemmell NJ (2010). The use of telomere length in ecology and evolutionary biology.. Heredity (Edinb).

[22] Shen M, Cawthon R, Rothman N, Weinstein SJ, Virtamo J (2011). A prospective study of telomere length measured by monochrome multiplex quantitative PCR and risk of lung cancer.. Lung Cancer.

[23] Strandberg TE, Saijonmaa O, Tilvis RS, Pitkala KH, Strandberg AY (2011). Association of telomere length in older men with mortality and midlife body mass index and smoking.. J Gerontol A Biol Sci Med Sci.

[24] Willeit P, Willeit J, Mayr A, Weger S, Oberhollenzer F (2010). Telomere length and risk of incident cancer and cancer mortality.. JAMA.

[25] Mannino DM, Buist AS (2007). Global burden of COPD: risk factors, prevalence, and future trends.. Lancet.

